# The function and clinical implication of YTHDF1 in the human system development and cancer

**DOI:** 10.1186/s40364-023-00452-1

**Published:** 2023-01-17

**Authors:** Wenjun Ren, Yixiao Yuan, Yongwu Li, Luciano Mutti, Jun Peng, Xiulin Jiang

**Affiliations:** 1 Department of Cardiovascular Surgery, The First People’s Hospital of Yunnan Province/The Affiliated Hospital of Kunming University of Science and Technology, Kunming, Yunnan China; 2 Key Laboratory of Molecular Oncology and Epigenetics, The First Affiliated Hospital of Chongqing Medical University, Chongqing, China; 3 Sbarro Institute for Cancer Research and Molecular Medicine, Center for Biotechnology, College of Science and Technology, Temple University, Philadelphia, PA 19122 USA; 4 Department of Biotechnological and Applied Clinical Sciences, University of L’Aquila, Via Vetoio, Coppito 2 67100 L’Aquila, Italy; 5 Kunming College of Life Science, University of Chinese Academy of Sciences, Beijing, 100049 China

**Keywords:** YTHDF1, Embryonic development, Stem cell, Self-renewal, Tumor, Biomarker

## Abstract

YTHDF1 is a well-characterized m6A reader protein that is essential for protein translation, stem cell self-renewal, and embryonic development. YTHDF1 regulates target gene expression by diverse molecular mechanisms, such as promoting protein translation or modulating the stability of mRNA. The cellular levels of YTHDF1 are precisely regulated by a complicated transcriptional, post-transcriptional, and post-translational network. Very solid evidence supports the pivotal role of YTHDF1 in embryonic development and human cancer progression. In this review, we discuss how YTHDF1 influences both the physiological and pathological biology of the central nervous, reproductive and immune systems. Therefore we focus on some relevant aspects of the regulatory role played by YTHDF1 as gene expression, complex cell networking: stem cell self-renewal, embryonic development, and human cancers progression. We propose that YTHDF1 is a promising future cancer biomarker for detection, progression, and prognosis. Targeting YTHDF1 holds therapeutic potential, as the overexpression of YTHDF1 is associated with tumor resistance to chemotherapy and immunotherapy.

## Background

RNA methylation is an important epigenetic modification in eukaryotic cells and plays a pivotal role in systems development and disease progression [1, 2]. RNA methylation modification is a dynamic biological process, which mainly involves three different components, including the “writers”, “erasers,” and “reader,” [3–5]. M6A reader protein recognizes the modification sites on its downstream target genes to produce different biological effects, including RNA splicing, regulation, nucleo-cytoplasmic transport, stability, translation, and degradation [6–11].

Among m6A regulators, YTHDF1 is the most abundant m6A reader that functionally connects m6A-modified mRNA to its eventual fate, mostly notably protein translation [12]. Different reading proteins play different biological functions after recognizing m6A modification. For instance, YTHDC1 acts as RNA splicing and nuclear export protein and leads to its unique localization and enrichment in the nucleus [13–15]. YTHDC2 plays a crucial role in RNA translation and decay when m6A-modified mRNA is recruited to processing bodies [16–18]. YTHDF3 enhances mRNA translation with the help of YTHDF1 and boosts the decay of m6A-modified transcripts mediated by YTHDF2 [19–21] and, as a well-characterized m6A reader protein. YTHDF1 is essential for protein translation, embryonic development, and stem cell self-renewal [22–24].

YTHDF1 regulates target gene expression by diverse molecular mechanisms, such as promoting protein translation or modulating the stability of mRNA [21, 25–27]. The cellular levels of YTHDF1 are precisely regulated by a complicated network at the levels of transcription, post-transcription, and post-translation. Therefore, in this review, we focus on three main aspects of YTHDF1: (a) genetic and biological cell machinery regulation. (b) stem cell self-renewal in central nervous, reproductive, and immune system development, and embryonic development, (d) human cancers progression. We propose that YTHDF1 is a promising future cancer biomarker for detection, progression, and prognosis.

### Composition of m6A

**Fig. 1:**
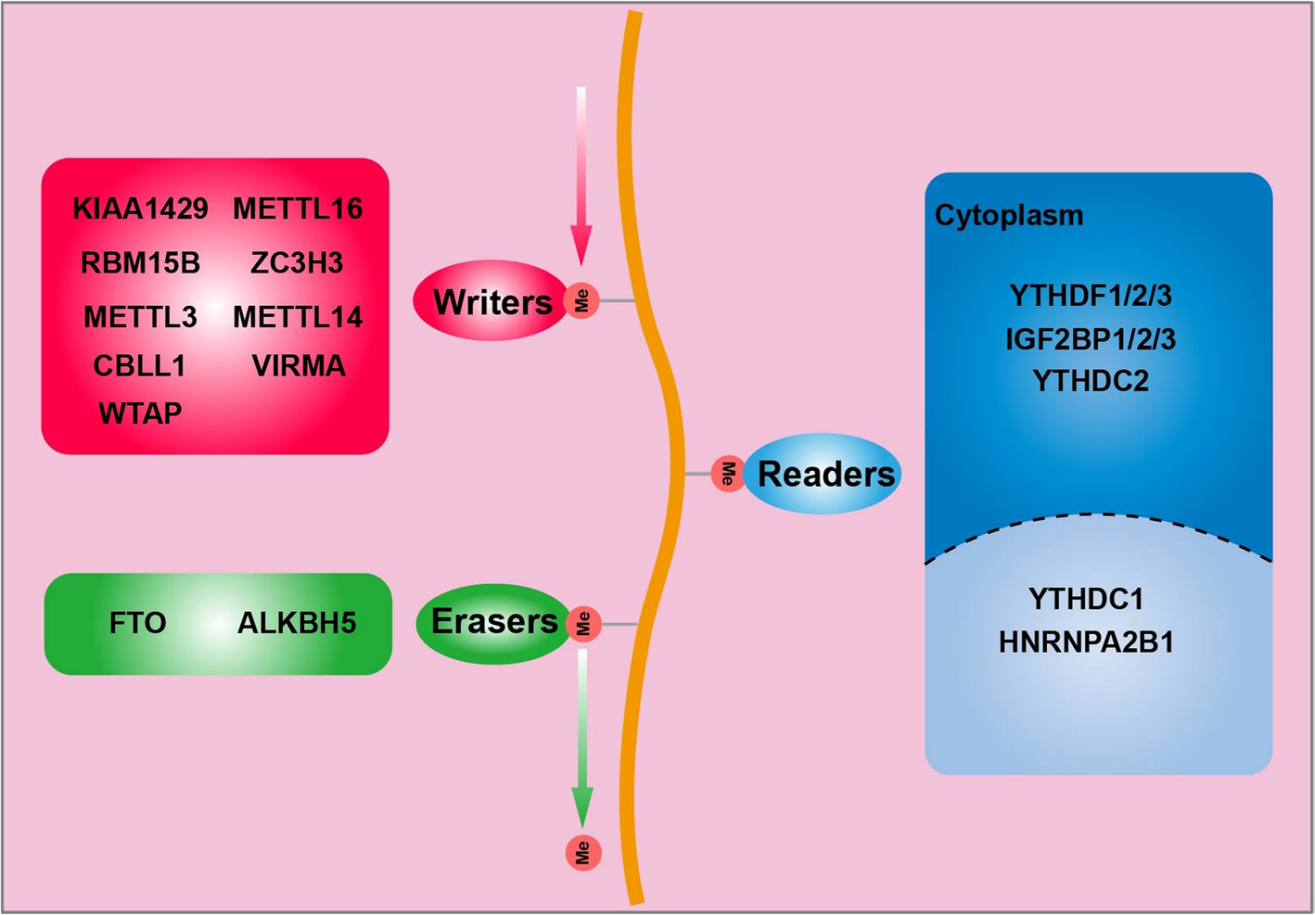
The composition and function of m6A modification. The m6A modification is installed by writers, including METTL3, METTL14, METTL16, CBLL1, WTAP, RBM15B, ZC3H3, VIRMA and KIAA1429. FTO and ALKBH5 are m6A erasers that remove m6A modifications. The m6A-modified RNA reader proteins (YTHDF1/2/3, YTHDC1/2, IGF2BP1/2/3, and HNRNPC/A2B1) are required to recognize m6A and regulate the RNA metabolism

M6A modification is a dynamic and reversible biological process, which requires writers, erasers, and readers **(**Fig. 1**)**. M6A is catalyzed by methyltransferase complex, which includes METTL3 [28], METTL14 [29], WTAP [30], KIAA1429 [31], METTL16 [32], RBM15 [33], and ZC3H13 [34]. The m6A is removed by demethylases such as FTO [35] and ALKBH5 [36]. The m6A erasers promote the transformation of m6A into N6-hydroxymethyladeosine and N6-forms adenosine successively, which is finally hydrolyzed into adenosine. The m6A reader proteins can recognize the m6A-modified RNAs and modulate RNA metabolism, which is divided into different protein families. One class of direct m6A readers proteins contain the YT521-B homology (YTH) domain, including YTH domain family 1–3 (YTHDF1–3) and YTH domain containing 1–2 (YTHDC1–2) in humans [2]. Several heterogeneous nuclear ribonucleoproteins (HNRNPs) fall into the other category, including HNRNPC, HNRNPG, and HNRNPA2B1, which mainly regulate alternative splicing or processing of target transcripts [37]. IGF2 mRNA binding proteins (IGF2BP1/2/3) families [38], and eukaryotic initiation factor (eIF) 3, belong to another subfamily member [39]. Numerous studies have shown that YTHDF1 plays an important role in tumor biology and nontumor lesions by mediating the protein translation of important genes or by affecting the expression of key factors involved in many important cell signaling pathways [14, 40–45].

### The role of YTHDF1 in the regulation of gene expression

#### YTHDF1 promotes translation

YTHDF1 promotes its target gene expression via recognition of m6A-edited mRNA and promotion of its translation [46]. However, there are diverse views on the mechanism by which it promotes protein translation. The previous study showed that YTHDF1 promotes translation initiation and subsequent protein translation [47]. For example, Su et al. found that YTHDF1 promotes the formation of the eIF3b translation initiation complex and subsequent YAP translation with depletion of eIF3b or YTHDF1 and the inhibition by METTL3-mediating translation events process [48]. The 5′ UTR region cap structure of eukaryotes helps improve translation efficiency. mRNA translation of JAK2 was mediated by YTHDF1.26 Rapamycin, an inhibitor of cap-dependent protein translation can markedly inhibit the increase of JAK2 protein expression promoted by YTHDF1, which indicates that a cap-dependent mechanism may be involved in YTHDF1-mediated translation [49]. Interestingly, a recent study found that YTHDF1 and YTHDF3 could synergistically promote protein translation efficiency. YTHDF1 and YTHDF3 complexes are paramount to recognizing mRNAs containing m6A modifications, thus promoting the translation of these mRNA. Consistently knockdown of YTHDF1 or YTHDF3 reduces the translation of the m6A-modified transcript [50]. In conclusion, the above results confirmed that YTHDF1 can promote translation by triggering translation initiation and elongation.

#### YTHDF1 enhances the stability of RNA

Several reports indicated that YTHDF1 plays a critical role in maintaining RNA stability. Wu et al. found that YTHDF1 elevates the stability of c-Myc mRNA catalyzed by METTL3 and promotes c-Myc expression [51]. Another study found that the knockdown of YTHDF1 significantly reduces the half-life of HK2 mRNA, suggesting that YTHDF1 could maintain the RNA stability of HK2 [52]. Additionally, YTHDF1 could combine eEF-2 and IGF2BP3 to promote PDK4 translation elongation and mRNA stability by binding with the m6A-modified 5′ UTR of its mRNA [53].

#### YTHDF1 is regulated at multi-levels

**Fig. 2:**
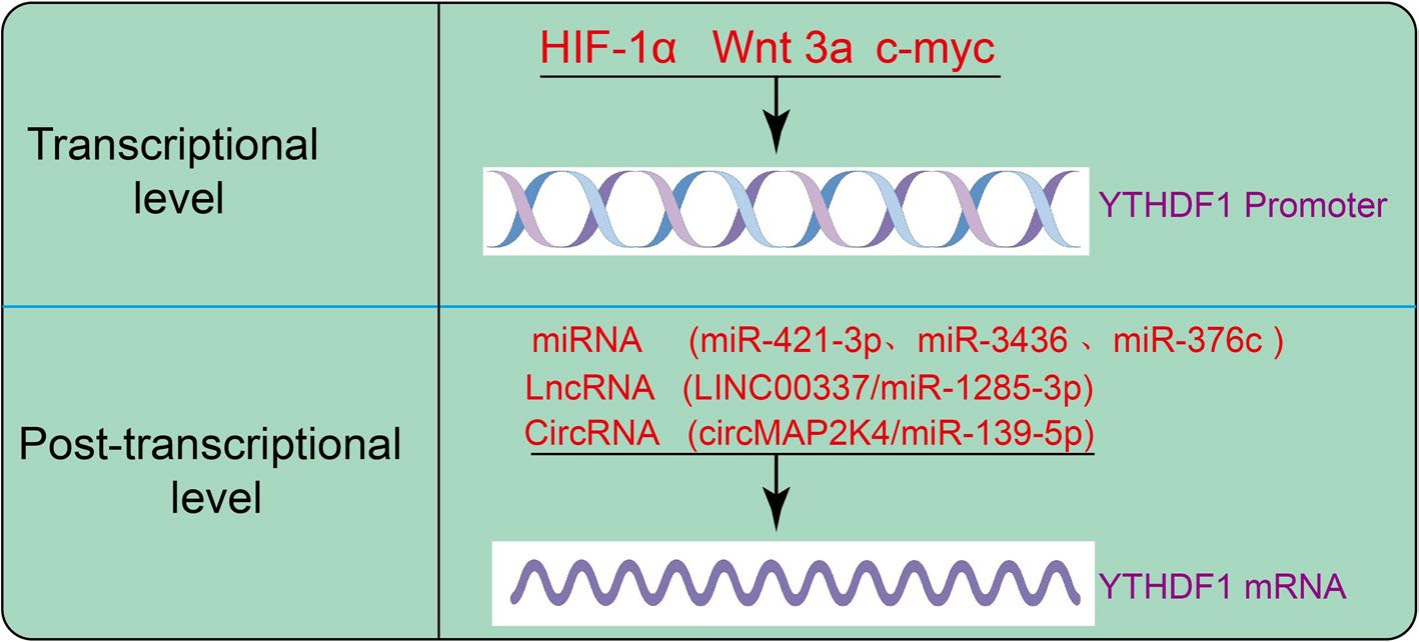
YTHDF1 is regulated at multiple levels. The YTHDF1 mRNA is regulated by transcription. The post-transcriptional regulators are mainly the miRNAs, lncRNAs, and circRNAs, which control the stability of YTHDF1 mRNA

Considering the critical role of YTHDF1 in gene expression, emerging studies have paid emphasis on the upstream regulatory mechanisms involved in the aberrant expression of YTHDF1 in various diseases **(**Fig. 2**)**. Previous studies have shown that transcription factors play a crucial role in regulating gene expression. For instance, Li et al., revealed that YTHDF1 plays a critical role in mediating protective autophagy in HCC cells, thereby allowing tumor cells to survive under the hypoxic tumor microenvironment [54]. Utilizing m6A-seq, proteomics, and polysome profiling, the authors showed that YTHDF1 promotes the translation of m6A-modified autophagy-related genes (ATG)-2A and ATG14, thus facilitating the induction of autophagy in HCC cells [55]. Wnt3a induces YTHF1 over-expression and promotes oxaliplatin-induced neuropathic pain in mice [56]. In colorectal cancer, the oncogenic transcription factor c-Myc promotes the expression of YTHDF1 and plays a significant role in colorectal cancer progression [57]. Besides, microRNA-421–3p prevents inflammatory response in cerebral ischemia/reperfusion injury by targeting m6A Reader YTHDF1 to inhibit p65 mRNA translation [58]. Similarly, miR-343 is involved in the regulation of YTHDF1 expression in glioma [59]. In NSCLC, miRNA-376c is down-regulated, while, on the contrary, YTDHF1 is highly expressed. Loading microRNA-376c in extracellular vesicles inhibits properties of non-small cell lung cancer cells by targeting YTHDF1 [54]. LncRNAs play an additional important role in regulating YTHDF1 expression. For example, in lung adenocarcinoma (LUAD) cells, elevated lncRNA LINC00337 expression significantly promotes YTHDF1 expression by sponging the miR-1285-3p thus promoting LUAD cells proliferation and metastasis [60]. In addition, circMAP2K4 positively modulates the YTHDF1 expression by restraining the expression of miR-139-5p and promoting HCC cell proliferation [61]. Taken together, these findings confirmed that YTHDF1 dysregulation can be affected by a series of complex molecular mechanisms. Investigation of the upstream regulatory mechanisms of YTHDF1 dysregulation helps us to better comprehend the biological role of YTHDF1 in cancers and provides possible therapeutic targets for anti-tumor therapy.

### The role of YTHDF1 in the physiological process

**Fig. 3:**
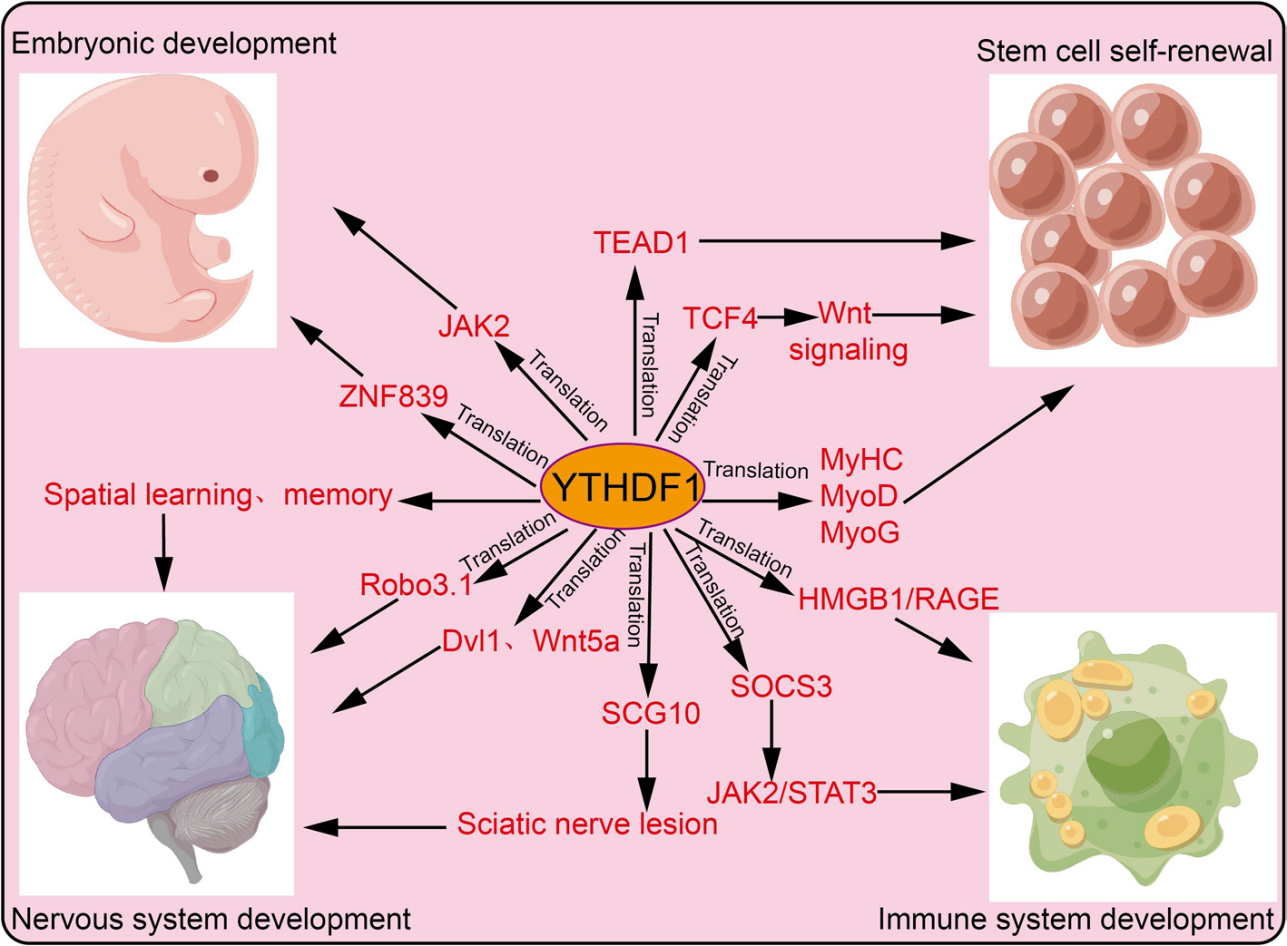
The target gene and functions of YTHDF1 in embryonic development, stem cell self-renewal, nervous system development, and immune system development. YTHDF1 recognizes m6A-edited mRNA and facilitates its translation, thus playing a critical role in embryonic development, stem cell self-renewal, nervous system development, and immune system development

Numerous studies confirmed that YTHDF1 plays crucial roles in stem cell self-renewal and embryonic development, central nervous and immune systems, we decided to mainly summarize and discuss how YTHDF1 influences both the physiological and pathological progressions in the above three systems (Fig. 3 and Table 1**)**.

**Table 1:** The function of YTHDF1 in normal system development

Embryonic/stem cell	Target gene	Functions	References
	JAK2	Promotes pluripotency of embryonic stem cells	[49]
	None	Promotes cardiomyocytes differentiation	[62]
	TEAD1	Promotes intestinal stemness	[63]
	TCF7L2/TCF4	Promotes intestinal stemness	[64]
	ZNF839	Promotes bone marrow mesenchymal stem cells	[65]
	MyHC/MyoD	Promotes Myogenic differentiation	[66]
	MAGED1	Promotes PASMC proliferation	[67]
Nervous system	None	Spatial learning and memory	[68]
	None	Promotes learning and memory	[25]
	robo3.1	Promotes pre-crossing axon guidance	[46]
	Dvl1/Wnt5a	Promotes axon growth	[69]
	SCG10	Promotes lesion-induced	[70]
Immune system	HMGB1/RAGE	Promotes immune paralysis	[71]
	SOCS3	Promotes anti-inflammatory	[72]
	Cathepsins	Promotes cross-presentation of tumor antigen	[24]

#### YTHDF1 regulates stem cell self-renewal and embryonic development

Stem cells possess replicating ability and multi-directional differentiation potential. Under certain conditions, they can differentiate into a variety of functional cells. According to the development stage of stem cells, they can be divided into embryonic stem cells and adult stem cells. Stem cells are primitive and unspecialized cells, which are not fully differentiated and have the potential of regenerating various tissues and organs. Because of their widespread presence within multicellular tissues, stem cells can progress into a variety of specialized cells through mitosis and differentiation, and generate more stem cells.

Studies indicated that YTHDF1 plays a critical role in regulating stem cell self-renewal. For instance, YTHDF1 was reported to enhance the translation efficiency of JAK2 which, in turn, promotes the pluripotency of embryonic stem cells [49]. A previous study identified that depletion of YTHDF1 significantly impairs cardiomyocyte differentiation and inhibits the expression of cardiomyocyte-specific genes [62]. Another study confirmed that YTHDF1 promotes intestinal cell stemness by recognition and translation of m6A-edited TEAD1 mRNA [63]. Similar results provided evidence that in intestinal stem cells, YTHDF1 is overexpressed via Wnt signaling and that knockdown of YTHDF1 inhibits the stemness of these cells.

Following activation by Wnt, YTHDF1 enhances the translation of TCF7L2/TCF4, thus maintaining their intestinal stem cell population during regeneration [64]. YTHDF1 is highly expressed in human bone marrow mesenchymal stem cells (hBMSCs) whereas depletion of YTHDF1 inhibits osteogenic cell differentiation. Interestingly, YTHDF1 modulates the translation of ZNF839 and ZFP839, resulting in the acceleration of the transcription activity of Runx2-driven osteogenesis of hBMSC [65]. YTHDF1 also promotes proliferation and myogenic differentiation by recognition and increased translation of m6A-edited MyHC, MyoD, and MyoG [66]. It has been shown that YTHDF1 was up-regulated in human and rodent pulmonary hypertension samples, knockdown of YTHDF1 ameliorated PASMC proliferation, phenotypic switching, and pulmonary hypertension development via promoting the translation of MAGED1 both in vivo and in vitro [67]. Taken together, YTHDF1 plays an essential role in embryonic development and stem cell self-renewal.

#### YTHDF1 regulates the nervous system-related physiological process

The central nervous system (CNS) is the main part of the nervous system, including the spinal cord and brain. Within this system, a large number of nerve cells form a network to transmit, store and process information, generate various psychological activities, and control all animal behaviors. YTHDF1 was up-regulated in the hippocampus of mouse brains and plays a crucial role in spatial learning and memory [68]. YTHDF1 also promotes learning and memory by enhancing the protein translation efficiency of targeted m6A-modified transcripts, under neuronal stimuli [25]. More importantly, a recent study found that YTHDF1 facilitates robo3.1 translation thus driving pre-crossing axon guidance in the spinal cord [46]. Ji et al. found that YTHDF1 is down-regulated in cerebellar granule cells and their axons whereas depletion of YTHDF1 significantly increases the axon growth rates of granule cells in vitro. Consistently knockdown of YTHDF1 in granule cells promotes parallel fiber growth, synapse formation in the cerebellum, and motor coordination ability. Further studies showed that YTHDF1 promotes the translation of Dvl1 and Wnt5a, leading to the activation of the Wnt pathway in cerebellar granule cells [69]. Another study showed that YTHDF1 plays an important role in sciatic nerve lesion-induced global protein synthesis and robust axon regeneration of dorsal root ganglion neurons. Consequently, the extension of regenerating SCG10+ axons was substantially decreased in YTHDF1 knockout mice [70].

#### YTHDF1 regulates the immune system-related physiological process

The immune system is paramount to maintaining the stability of the environment and physiological balance in the body. It has been shown that depletion of YTHDF1 significantly reduces macrophages, Th1/Th17, and CTL dysfunction together with macrophage-dependent endothelial damage via HMGB1/RAGE [71]. Another study found that infection by bacterium treponema pallidum significantly increases the translation of macrophagic YTHF1 that recognizes and binds to the m6A methylated-SOCS3 mRNA thus decreasing the secretion of inflammatory factors via JAK2/STAT3 pathway inhibition to control the anti-inflammatory response [72]. Knockdown of YTHDF1 promotes the cross-presentation of tumor antigen and the cross-priming of CD8+ T cells via translation of lysosomal cathepsins in Dendritic Cells whereas inhibition of cathepsins significantly increases cross-presentation by wild-type DCs [24].

### The role of YTHDF1 in Cancer

**Fig. 4:**
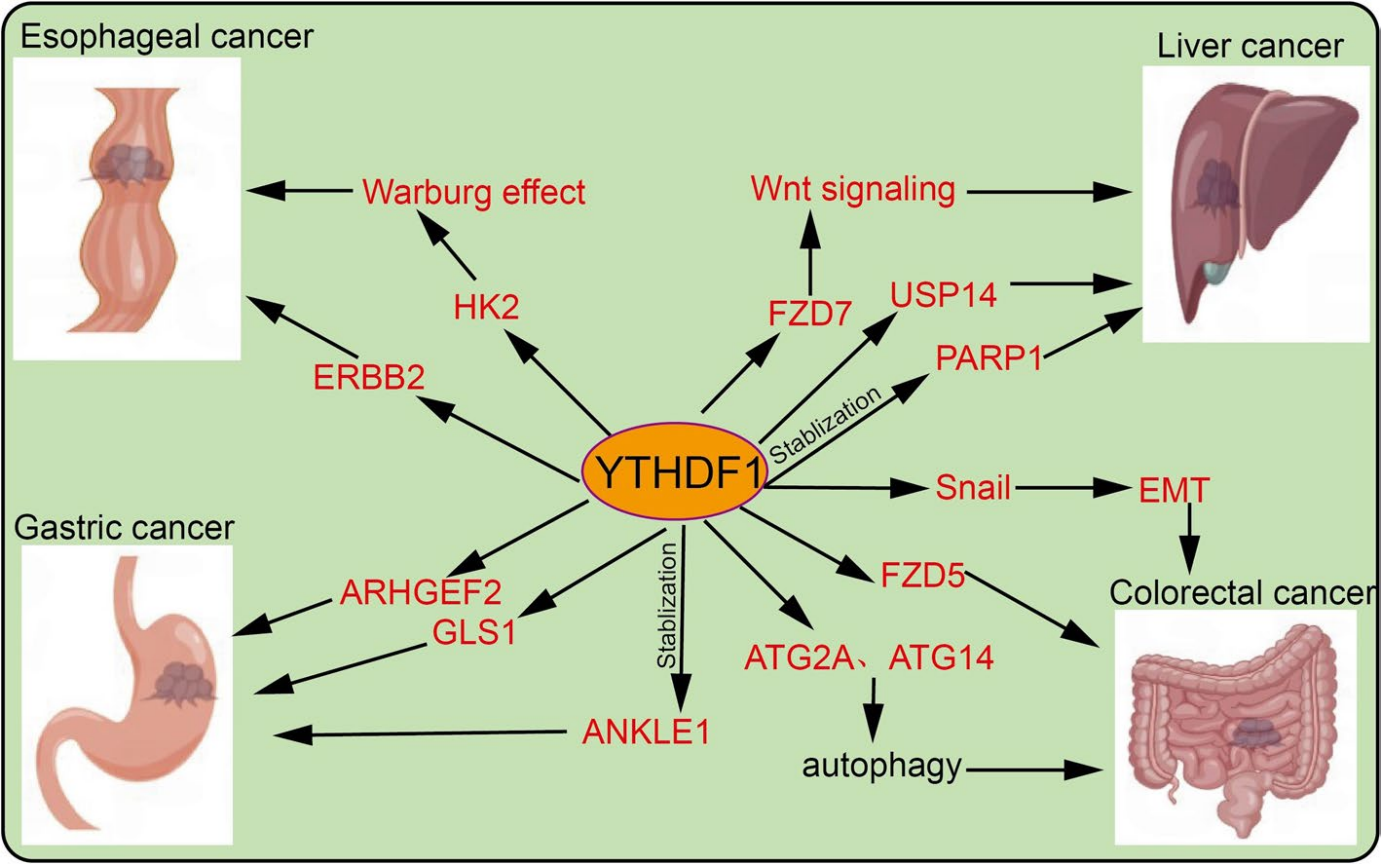
The target gene and functions of YTHDF1 in esophageal cancer, gastric cancer, colorectal cancer, and liver cancer. YTHDF1 recognizes m6A-edited mRNA and facilitates its translation, thus playing a critical role in the progression of esophageal cancer, gastric cancer, colorectal cancer, and liver cancer

**Fig. 5:**
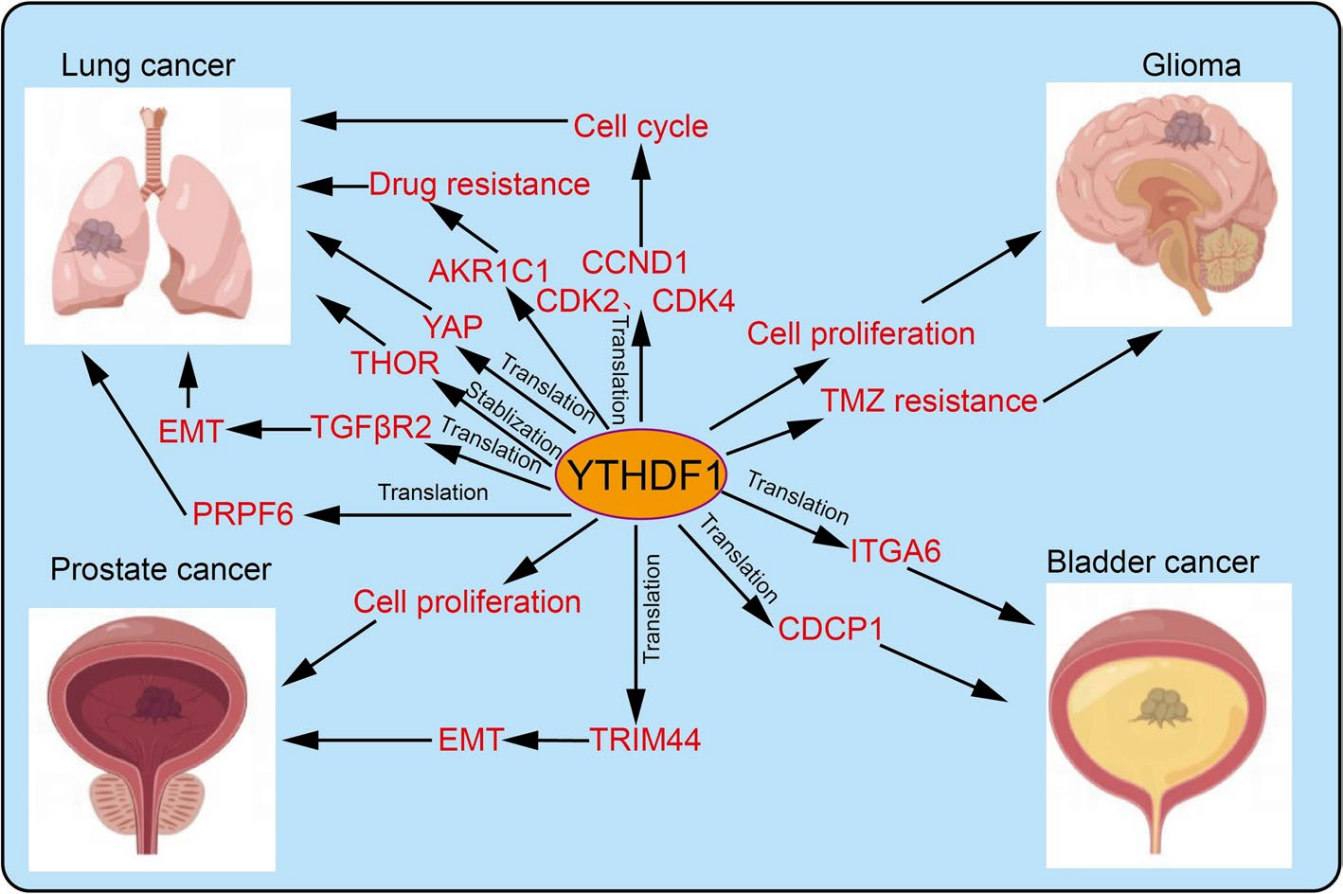
The target gene and functions of YTHDF1 in lung cancer, glioma, prostate cancer, and bladder cancer. YTHDF1 recognizes m6A-edited mRNA and facilitates its translation, thus playing a critical role in the progression of lung cancer, glioma, prostate cancer, and bladder cancer

**Fig. 6:**
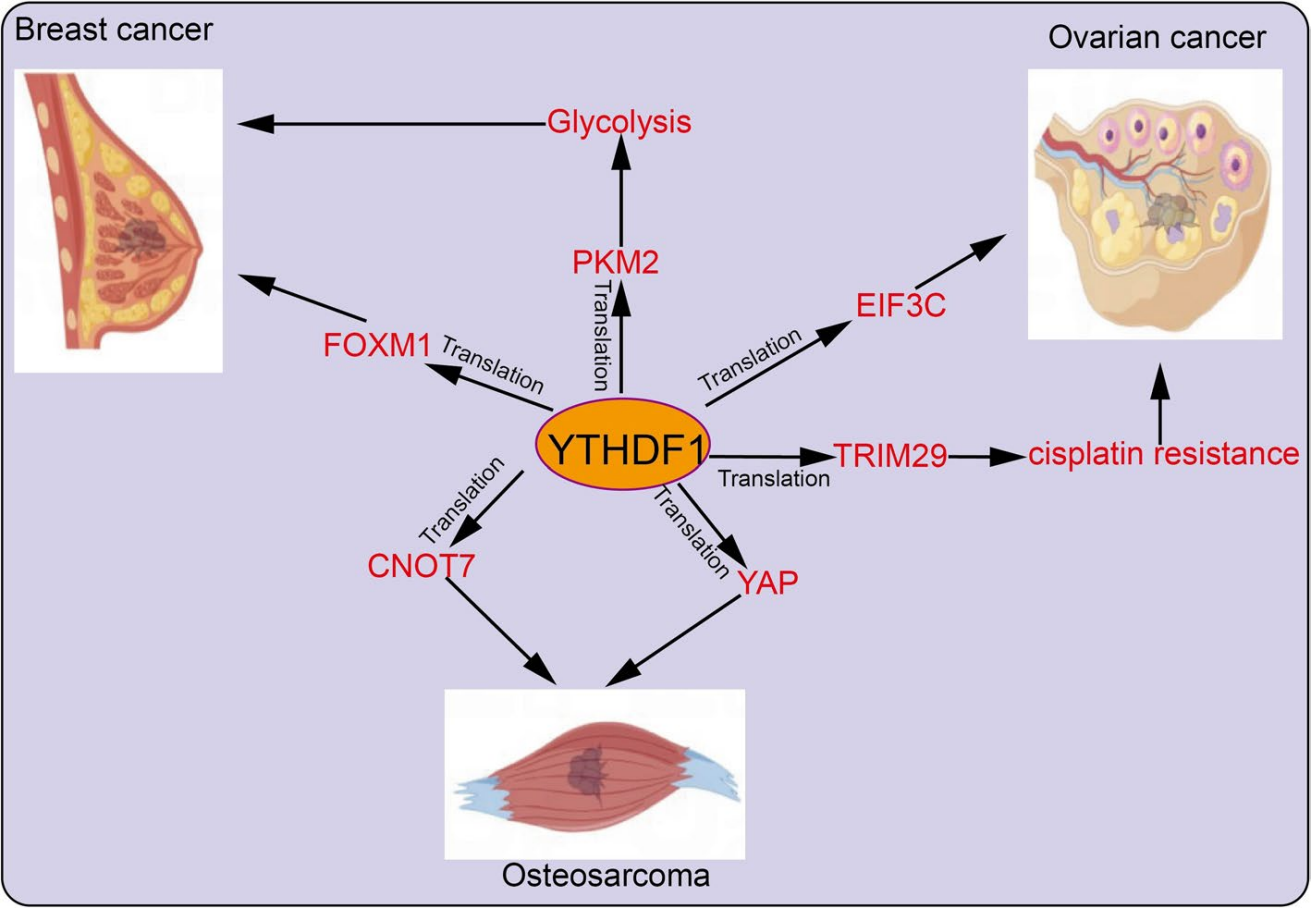
The target gene and functions of YTHDF1 in breast cancer, ovarian cancer, and osteosarcoma. YTHDF1 recognizes m6A-edited mRNA and facilitates its translation, thus playing a critical role in the progression of breast cancer, ovarian cancer, and osteosarcoma

Overwhelming evidence substantiates that dysregulation of YTHDF1 is tightly correlated with the malignant progression of various cancer. Therefore, we systematically summarize the recent advances of YTHDF1 in human cancer (Figs. 4, 5, 6 and Table 2).

**Table 2:** The function of YTHDF1 in human cancers

Cancer Type	Regulation	Target gene	Roles	Function	References
Esophageal cancer	Up	HK2	Translation	Promotes cell proliferation	[73]
Esophageal cancer	Up	ERBB2	Stabilization	Promotes cell proliferation/migration	[74]
Gastric cancer	Up	FZD7	Translation	Promotes cell proliferation/migration	[23]
Gastric cancer	Up	USP14	Translation	Promotes cell proliferation/migration/EMT	[75]
Gastric cancer	Up	PARP1	Translation	Promotes oxaliplatin resistance	[76]
Colorectal cancer	Up	ARHGEF2	Translation	Promotes EMT	[77]
Gastric cancer	Up	GLS1	Translation	Promotes cell proliferation/migration	[78]
Gastric cancer	Up	ANKLE1	Translation	Promotes cell proliferation	[79]
Liver cancer	Up	Snail	Translation	Promotes EMT	[80]
Liver cancer	Up	FZD5	Translation	Promotes cell proliferation/migration	[81]
Liver cancer	Up	ATG2A	Translation	Promotes autophagy, growth, and metastasis	[55]
Lung cancer	Up	CDK2	Translation	Promotes cell proliferation	[22]
Lung cancer	Up	YAP	Translation	Promotes cell proliferation/migration/EMT	[82]
Lung cancer	Up	THOR	Stabilization	Promotes cell proliferation	[83]
Lung cancer	Up	TGFβR2	Translation	Promotes EMT	[84]
Lung cancer	Up	PRPF6	Translation	Promotes cell proliferation/migration	[85]
Bladder cancer	Up	ITGA6	Translation	Promotes cell proliferation/migration	[50]
Bladder cancer	Up	CDCP1	Translation	Promotes cell proliferation/migration	[86]
Ovarian cancer	Up	EIF3C	Translation	Promotes cell proliferation	[87]
Ovarian cancer	Up	TRIM29	Translation	Promotes cisplatin-resistance	[88]
Breast cancer	Up	PKM2	Translation	Promotes glycolysis	[89]
Breast cancer	Up	FOXM1	Translation	Promotes cell proliferation/migration	[90]
Osteosarcoma	Up	CNOT7	Translation	Promotes cell proliferation/migration/EMT	[91]
Osteosarcoma	Up	YAP	Translation	Promotes cell proliferation/migration/EMT	[92]

### Esophageal cancer

In esophageal cancer (ESC), YTHDF1 is highly expressed and correlated with multiple adverse clinical features [93]. LncRNA-HCP5 directly interacts with YTHDF1 and promotes m6A-modified HK2 mRNA translation, thereby boosting the Warburg effect of ESC cells, and ESC progression [73]. Similarly, YTHDF1 promotes ESC progression via the stabilization of ERBB2 mRNA via recognition of m6A modification [74].

### Gastric cancer

Yu et al. demonstrated that in gastric cancer (GC) YTHDF1 is up-regulated and related to aggressive tumor progression and poor overall survival. Knockdown of YTHDF1 inhibits GC cell proliferation and tumor growth whereas YTHDF1 promotes GC progression via translation of FZD7 in an m6A-dependent manner and activation of the Wnt/β-catenin pathway [23]. Zhu et al. found that YTHDF1 accelerates the tumorigenesis and metastasis of GC via recognition of m6A-edited USP14 mRNA and its protein expression [75]. Zhang et al. demonstrated that YTHDF1 promotes oxaliplatin resistance to gastric cancer maintaining the stability of PARP1 in an m6A-dependent manner [76].

### Colorectal cancer

YTHDFs is highly expressed in colorectal cancer (CRC) and associated with metastatic disease via increased translation of ARHGEF2 mRNA [94]. On the other hand, another study revealed that YTHDF1 exerts a pro-tumorigenic effect by recognizing and promoting the translation of m6A-modified FZD9 and WNT6 mRNA, leading to the aberrant activation of WNT/β-catenin signaling and ultimately promoting tumorigenicity and stem cell-like activity in CRC [77]. Additionally, YTHDF1 was found to rewire tumor metabolism by promoting the protein translation of glutaminase 1 (GLS1) by targeting the 3′UTR of GLS1 mRNA, which contributes to chemoresistance to cisplatin [78]. YTHDF1 also enhances the transcriptional efficiency of ANKLE1 however it does not maintain ANKLE1 mRNA stability, via recognition of 6A modification thus acting as a tumor suppressor and playing a crucial role in the inhibition of colorectal cancer cell proliferation [79].

### Liver cancer

In liver cancer (HCC), YTHDF1 plays a central role in modulating the cell cycle and cell metabolism of the liver cell via tagging Snail, a crucial EMT-related transcription factor, which is essential for the metastasis of HCC cells [80]. Another study demonstrated that YTHDF1 promotes HCC progression via recognition and translation of m6A-modified FZD5 mRNA CDS [81]. Wang et al. reported that YTHDF1 is overexpressed in HCC, induced by hypoxia-related transcription factor HIF-1a, and is correlated with adverse clinical outcomes in HCC patients. YTHDF1 promotes autophagy-related malignancy of HCC via direct binding to m6A-modified ATG2A and ATG14 mRNA, thereby facilitating the translation of ATG2A and ATG14 [55]. Consistently, YTHDF1 knockdown significantly inhibits HCC autophagy, growth, and metastasis [55].

### Lung cancer

We have previous results showed that YTHDF1 promotes NSCLC cell proliferation and xenograft tumor formation by mediating the translational efficiency of cyclin D1, cyclin-dependent kinase 2 (CDK2), and CDK4, and that YTHDF1 elimination inhibits de novo lung adenocarcinoma (ADC) progression [22]. Similarly, another study found that YTHDF1 promotes cell growth, invasion, and EMT of NSCLC cells via interaction with eIF3b and increased YAP mRNA translation in m6A dependent manner [82]. Furthermore, YTHDF1 was found to promote lung cancer progression by reading m6A motifs of lncRNA THOR thus enhancing the stability of lncRNA THOR, [83]. Gao et al. demonstrated that LINC00337 is positively regulated by sponging YTHDF1, with the subsequent promotion of LUAD progression [60]. Zhang et al. found that YTHDF1-mediated TGFβR2 mRNA stabilization and increased expression promote the EMT of NSCLC [84]. It has been shown that YTHDF1 is up-regulated in 5-fluorouracil (5-FU) and oxaliplatin-resistant NSCLC cells whereas knockdown of YTHDF1 boosts the cisplatin resistance of NSCLC via modulates the Keap1-Nrf2-AKR1C1 axis [22]. YTHDF1 positive regulates the expression of small nuclear ribonucleic protein PRPF6 via direct recognition ad translation of m6A-edited PRPF6 in LUAD cells [85].

### Glioma and glioblastoma

YTHDF1 is elevated in glioblastoma (GBM) and positively regulated by MSI1 whereas both of these proteins are related to worse prognosis in glioblastoma patients [95]. Moreover, high YTHDF1 expression significantly increases in vivo proliferation and tumor growth of glioblastoma [59]. It is noteworthy that YTHDF1 knockdown promotes the sensitivity of glioblastoma cells on TMZ, a major drug for glioblastoma chemotherapy [95].

### Prostate and bladder cancer

YTHDF1 is overexpressed in prostate cancer tissues and cells. Patients with higher YTHDF1 expression show a worse prognosis whereas YTHDF1 knockdown significantly inhibits prostate cancer cells proliferation, migration, and invasion via modulating the expression of TRIM44 [96]. Hu et al. found that YTHDF1 is upregulated in prostate cancer and higher expression of YTHDF1 is associated with lymph node metastasis and higher Gleason grades in prostate cancer patients [97]. It has also been reported that YTHDF1 promotes cell growth and progression of bladder cancer via recognition of the m6A sites and translation of ITGA6 [50]. It has been confirmed that CDCP1 was overexpressed in bladder cancer cells, mediated by m6A methyltransferase METTL3, m6A reader YTHDF1 preferentially recognizes m6A residues on CPCP1 3′-UTR and enhances the CDCP1 translation, promotes bladder cancer tumorigenesis in vitro and in vivo [86].

### Ovarian Cancer

In addition to ovarian cancer, YTHDF1 is overexpressed in cancer tissues and is correlated with poor prognosis. Mechanistically, YTHDF1 promotes the translation of EIF3C by binding to m6A-modified EIF3C mRNA and concomitantly determines the overall translational output, thereby facilitating tumorigenesis and metastasis of ovarian cancer. EIF3C, as a subunit of the protein translation initiation factor EIF3, also promotes the translation of other proteins in cells [87]. TRIM29 is highly expressed in cisplatin-resistant ovarian cancer cells and correlates with poor prognosis in these patients. Mechanisms research indicated that YTHDF1 promotes the CSC-like characteristics of the cisplatin-resistant ovarian cancer cells via recognition of m6A-edited TRIM29 and increased TRIM29 translation [88].

### Breast Cancer

In breast cancer, studies show that hypoxia up-regulates the expression of YTHDF1 that promotes cell proliferation and invasion thus driving tumorigenicity and metastasis. These effects are mediated by the YTHDF1-regulated translation of PKM2 and the subsequent switch to the glycolytic process [89]. Similarly, YTHDF1 is elevated in breast cancer cells and clinical tissue specimens and related to tumor size, lymph node invasion, and distant metastasis in breast cancer patients. YTHDF1 knock-down inhibits breast cancer cell proliferation, and invasion, and induces G0/G1 phase cell cycle arrest. These effects depend on the property of YTHDF1 to recognize and bind to m6A-modified mRNA of FOXM1 leading to enhanced FOXM1 translation efficiency and promotion of breast cancer metastasis [90].

### Osteosarcoma

It is well known that YTHDF1 is overexpressed in osteosarcoma (OS) and correlated with the poor prognosis of these patients. Depletion of YTHDF1 suppresses the proliferation, migration, and invasion of the OS cells, via regulation of the expression of CNOT7 [91]. Similarly, YTHDF1 was reported to enhance the translation of methylated YAP transcripts and participate in the tumor progression of OS [92].

### Other cancers

Studies have confirmed that m6A RNA modification was dysregulated in PDAC tissues more than in adjacent normal tissues [98]. Huang et al. found that higher YTHDF1 expression correlates with a favorable prognosis and plays a tumor suppressor role in pancreatic ductal adenocarcinoma (PDAC) [99]. YTHDF1 has high copy gains and overexpression in Merkel cell carcinoma, YTHDF1 amplification was positively regulate the expression of MCPyV, and knockdown of YTHDF1 significantly inhibited the Merkel cell proliferation via down-regulate the translation initiation factor eIF3 [80].

### Clinical implications of YTHDF1 in human cancers

#### YTHDF1 as a potential biomarker in human cancer

In summary, most of the studies reported thus far indicate that YTHDF1 can be considered a promising cancer biomarker in a broad range of human tumors: high expression of YTHDF1 is an independent negative prognostic biomarker in patients with NSCLC [22], HCC [55], GC [75], Ovarian [87] and prostate cancer [96], cervical squamous cell carcinoma [100] and CRC [101]. Furthermore, it has been confirmed that elevated YTHDF1 expression is correlated with various malignant tumor behaviors, including invasiveness, and lymph node metastasis. Similarly, YTHDF1 is elevated in prostate cancer and breast cancer tissues, and its high expression is associated with nodal metastasis and lymph node metastasis together with poor prognosis respectively [102]. Similarly, up-regulation of YTHDF1 is positively associated with advanced tumor grade of glioma [59]. In summary, most of the studies reported thus far indicate that YTHDF1 is highly expressed in human cancers and correlates with poor prognosis, implying that YTHDF1 functions as an oncogenic factor.

#### The impact of YTHDF1 on chemotherapy

Chemoresistance usually results in treatment failure and is correlated with poor prognosis for cancer patients. Emerging work has shown that YTHDF1 plays an important role in cancer chemoresistance. For example, Nishizawa et al. found that YTHDF1, induced by c-Myc, was highly expressed in CRC cells, and knockdown of YTHDF1 could sensitize CRC cells to chemotherapeutic drugs fluorouracil and oxaliplatin [57]. YTHDF1-mediated glutamine metabolism via GLS1 has been shown to promote cisplatin chemoresistance in CRC cells, while the combination of YTHDF1 silencing and cisplatin leads to a synergistic effect in suppressing tumor growth [78]. Consistently, our recent study found that YTHDF1, as a hypoxia adaptation-related gene, is down-regulated in Tibetan domestic mammals compared to lowlanders [22]. Furthermore, the knockdown of YTHDF1 rendered cancer cells resistant to cisplatin treatment via the Keap1-Nrf2-AKR1C1 axis in NSCLC [22]. Therefore, targeting YTHDF1 may combat chemoresistance and alleviate the potential side effects of high-dose cisplatin [22].

#### The impact of YTHDF1 on immunotherapy

Emerging work has shown that immune checkpoint blockade (ICB) therapy is effective against advanced human cancer, however; only a small subset of cancer patients could benefit from anti-PD-1/PD-L1 immunotherapy [103]. Therefore, there is an urgent need to identify factors that can modulate ICB responses. A previous study identified that YTDHF1 appears to be significantly correlated with DCs in the tumor microenvironment. Genetic ablation of YTHDF1 in mice leads to reduced tumor growth associated with increased tumor infiltration by cytotoxic T cells, whilst simultaneously reducing infiltration of myeloid-derived suppressor cells (MDSC) [24]. Mechanistically, the knockdown of YTHDF1 promotes cross-presentation of tumor-associated antigens by DCs, which activate CD8+ T cell-mediated adaptive immune response. ICB therapeutic response is significantly enhanced in YTHDF1-knockout mice as compared to wild-type mice [24]. Loss of YTHDF1 in tumor cells leads to the recruitment of mature DCs in the tumor, which in turn promotes the infiltration of T helper cells and cytotoxic T cells, as well as the increased production of cytotoxic cytokines. These studies collectively confirmed that the targeting of YTHDF1 in human cancer could reactivate antitumor immunity and potentiate the therapeutic effect of ICB therapy.

## Discussions

YTHDF1 is an essential gene required for embryogenesis, organ development, and disease progression. The dynamic expression of YTHDF1 is strictly controlled by a complex regulatory network at multiple levels, including transcription, post-transcription, and post-translation, and its expression balance controls the expression of a downstream target gene that regulates several signaling pathways to participate in cancer progression. Understanding its responsible regulatory mechanism and modulation of its expression in different ways are extremely important prospects for the diagnosis and treatment of human diseases.

In terms of clinical application, YTHDF1 was reported to be an independent marker for the diagnosis and prognosis of human cancer. Accumulating evidence confirmed that YTHDF1 dysregulation correlated with radioresistance and poor clinical outcomes in patients with cancers. Genetic depletion of YTHDF1 sensitizes human tumors to chemotherapy and immunotherapy, suggesting that YTHDF1 antagonists can be potential adjuvants in cancer therapy. In particular, the targeting of YTHDF1 has been shown to mediate a switch from immunological “cold” tumors to “hot” tumors, and thus presents great potential in combination with ICB therapy. Collectively, targeting YTHDF1 represents a promising approach for the future management of human cancers.

YTHDF1 plays a paramount role in embryonic development which certainly imply its relevance also a potential target/biomarker in human physiology. However, there are many problems to be solved. First, m6A modification sites exist in different regions of RNA transcripts, including 3 UTR, CDS, and 5UTR therefore, identifying modifications at different sites may have different effects, regulating the translation of target genes or maintaining their stability. Furthermore, it is unknown which site is more important for its function, and so far there are no YTHDF1 small molecule inhibitors identified yet. Therefore, as described in this review, because of its crucial role in human progression it will be extremely important to develop small molecule inhibitors of YTHDF1 or screen natural compounds that can down-regulation YTHDF1 for cancer treatment.

## Conclusions

In summary, YTHDF1 plays a critical role in both human physiological system development and human cancer progression, and it may serve as a promising diagnostic/prognostic biomarker and a potential therapeutic target.

## Data Availability

Not applicable.

## Abbreviations

3’UTRs: 3′ Untranslated regions
CRC: Colorectal cancer
eIF: Eukaryotic initiation factor
EMT: Epithelial-mesenchymal transition
FTO: Fat mass and obesity-associated protein
IGF2BP: IGF2 mRNA binding protein
m6A: N6-methyladenosine
METTL3/14/16: Methyltransferase-like 3/14/16
RBM15/15B: RNA-binding motif protein 15/15B
SOCS2: Suppressor of cytokine signaling 2
SRSF5: SR-like splicing factors 5
TCF4: Transcriptional factor 4
USP48: Ubiquitin specific peptidase 48
VIRMA/KIAA1429: Virlike m6A methyltransferase-associated
WTAP: WT1-associated protein
YTHD: YTH domain family of protein
ZC3H13: Zinc finger CCCH-type containing 13
